# Foreign bodies logded in the hypopharynx

**DOI:** 10.11604/pamj.2021.38.319.28312

**Published:** 2021-03-30

**Authors:** Miguel Carvalho, Rui Vieira

**Affiliations:** 1 Otolaryngology Department, Centro Hospitalar Tondela-Viseu, Viseu, Portugal,; 2 Anestesiology Department, Centro Hospitalar Tondela-Viseu, Viseu, Portugal

**Keywords:** Thumbtacks, pyriform sinus, valeculae

## Image in medicine

We present the case of a 21-year-old female, with a history of bipolar disease, observed at the emergency department of our institution for voluntary ingestion of two foreign bodies. She complained of localized neck pain, dysphagia and odynophagia that started 2 hours before observation. Initial evaluation identified two thumbtacks lodged in the left pyriform sinus and valeculae. Despite swift preparation for removal of the foreign bodies under sedation, progression on the digestive tube occurred. One thumbtack was removed by endoscopic techniques from the gastric fundus. The other was expelled naturally in an uncomplicated fashion at day 8.

**Figure 1:**
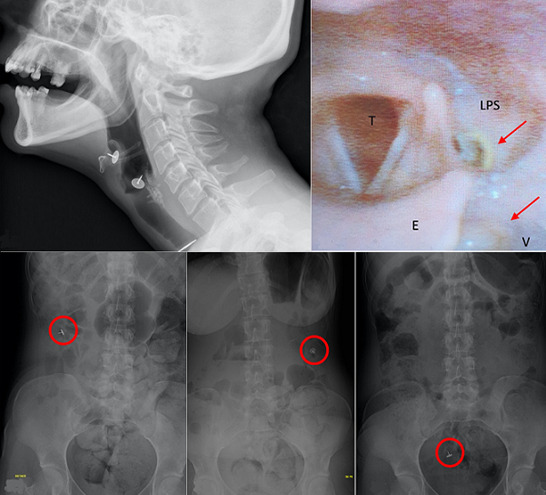
A) lateral neck x-ray; two foreign bodies are identified (thumbtacks); B) fibroscopic evaluation; C) progression of the thumbtack not removed endoscopically (day 2, 5 and 7 respectively) (E: epiglotis; LPS: left pyriform sinus; T: trachea; V: valeculae)

